# Comprehensive quality profiling and multivariate analysis of rice (*Oryza sativa* L.) cultivars: integrating physical, cooking, nutritional, and micronutrient characteristics for enhanced varietal selection

**DOI:** 10.1186/s12870-025-06438-5

**Published:** 2025-04-17

**Authors:** Khaled M. H. Abdelsalam, Ahmed M. Shaalan, Germine M. AbouEl-Soud, Medhat A. E. El-Dalil, Abdelsalam M. Marei, Diaa Abd El-Moneim, Aly A. A. El-Banna, Sobhi F. Lamlom, Ahmed M. Abdelghany

**Affiliations:** 1 Rice Technology Training Center (RTTC), Field Crops Research Institute, Agricultural Research Center, Alexandria, Egypt; 2 Plant Production Department, Faculty of Desert and Environmental Agriculture, Matrouh University, Matrouh, 51744 Egypt; 3 Department of Plant Production, (Genetic Branch), Faculty of Environmental and Agricultural Sciences, Arish University, El-Arish, Egypt; 4 Plant Production Department, Faculty of Agriculture Saba Basha, Alexandria University, Alexandria, 21531 Egypt; 5 Crop Science Department, Faculty of Agriculture, Damanhour University, Damanhour, Egypt

**Keywords:** Rice quality traits, Multivariate analysis, Nutritional profiling, Grain characteristics, Cooking properties, Varietal selection

## Abstract

**Background:**

Rice (*Oryza sativa* L.) is a staple food for nearly half the global population, with rice grain quality (RGQ) and yield being the most valuable attributes for consumers and food security. RGQ encompasses multiple interconnected features including physical appearance, cooking properties, biochemical composition, nutritional components, and sensory aspects.

**Methods:**

This study evaluated the agronomic performance of four commercial rice cultivars (Giza 178, Sakha 108, Sakha Super 300, and Egyptian Yasmin) during the 2022 and 2023 growing seasons. The experiment was conducted at the Rice Technology Training Center in Alexandria using a randomized complete block design with three replications. A cultivars were selected based on their commercial significance and diverse genetic backgrounds to represent the primary rice varieties grown in Egypt.

**Results:**

Analysis of variance revealed significant genotypic effects (*p* < 0.001) for most traits, with notable genotype × environment interactions in milling quality and water uptake characteristics. Multivariate analyses, including Principal Component Analysis (PCA), hierarchical clustering, and correlation analysis, provided complementary evidence for cultivar differentiation. PCA demonstrated that 94.2% of total variance was explained by two principal components, with Yasmin distinctly clustering in the positive quadrant of Dim1, showing superior performance in nutritional and cooking parameters (protein: 8.51%, fiber: 0.33%, water uptake: 439.45%, elongation: 60.73%). Hierarchical cluster analysis revealed two distinct trait groupings: physical-processing parameters and nutritional-functional attributes. Cultivar Super 300 demonstrated superior performance in physical-processing metrics (milling yield: 71.69%, grain hardness: 6.56), while Yasmin exhibited exceptional nutritional-functional characteristics. Furthermore, correlation analysis revealed significant relationships among quality parameters (*p* < 0.001), particularly between physical characteristics and milling traits (*r* = 0.99), and among nutritional components (*r* = 0.87–0.99).

**Conclusion:**

The integrated multivariate approach identified Yasmin as the superior cultivar for nutritional and cooking qualities, while Super 300 excelled in physical parameters, providing comprehensive insights for developing cultivars with optimized quality profiles tailored to specific market demands and consumer preferences.

## Introduction

Rice (*Oryza sativa* L.) is a key component of global food security, providing essential nutrition to more than fifty percent of the world’s population. In Egypt, rice production holds significant importance with a remarkable national output of 5.6 million tons across 642,501 hectares of harvested land, resulting in impressive yields of 8.72 ton/ha [1]. The increasing demand for high-quality rice varieties, coupled with evolving consumer preferences, has intensified the need for comprehensive quality assessment methods in rice breeding and selection programs [2, 3].

Rice is a significant source of protein, including eight necessary amino acids in balanced ratios, which supports good hair, skin, cardiovascular and pulmonary health, as well as optimal nervous system performance [4]. Rice generally comprises approximately 7% protein, which is inferior to the protein content found in pulses and wheat [5]. Despite rice’s significant role in cereals, there has been minimal effort to enhance its protein content. Nonetheless, increasing protein content in rice helps mitigate malnutrition in nations where rice is a primary staple, fulfilling daily caloric needs while lacking various critical elements. Recently, researchers have been looking into ways to increase the amount of micronutrients in rice grains through agronomic biofortification [6, 7]. These methods include conventional breeding, marker-assisted breeding, genetic engineering, and omics techniques that are meant to make rice healthier. This review concentrates on rice quality, namely its nutritional value and importance, while also examining several techniques to improve the nutritional quality of rice.

Rice grain quality (RGQ) and yield are the most sought-after and significant characteristics of rice in relation to customer desire and food security [8]. Recent technological improvements have enabled rice production to meet the demands of the expanding population, so contributing to the gradual improvement of living conditions, consumption levels, and individual preferences. Consequently, improving the grain quality of rice is becoming increasingly significant; however, existing rice varietal breeding initiatives have not sufficiently achieved this objective [9]. The evaluation and acceptability of RGQ are contingent upon the visual, olfactory, and gustatory perceptions of the end user, as well as their cultural, historical, and regional context [10]. RGQ is the principal determinant influencing the market value of rice in rice-consuming countries and is crucial for the adoption of novel varieties by both farmers and consumers [11]. The quality of rice grains is influenced by various complex and interrelated characteristics, such as their physical appearance, cooking and eating properties, biochemical composition, nutritional elements, and sensory attributes, which can be classified as intrinsic or extrinsic factors [12]. The inherent characteristics include head rice, grain morphology and dimensions, homogeneity, purity, tenderness, coloration, hygiene, and aroma. The external features encompass branding, packaging, and labeling [13]. Consequently, the phrase “rice quality” is all-encompassing and encompasses various critical product characteristics, from rice production to its consumption post-processing [14, 15].

Rice grain quality is evaluated using diverse traits and parameters that vary across countries, but four primary quality traits are commonly assessed: physical appearance, milling quality, cooking quality, and nutritional quality [16]. Enhancing the economic and nutritional value of rice requires a thorough understanding of the factors influencing the production of different rice varieties, including variety selection, harvesting conditions, and environmental factors such as light, temperature, humidity, and post-harvest management [6]. Rice is a vital source of nutrients, including calcium (Ca), iron (Fe), zinc (Zn), potassium (K), phosphorus (P), and sodium (Na), as well as essential vitamins and minerals [17]. However, compared to other staple crops like maize, wheat, and legumes, rice generally has lower mineral and vitamin B content [18]. Brown rice is nutritionally superior to white rice, as its bran layer contains protein, fatty acids, vitamins, minerals, and antioxidants [19]. Rice bran, in particular, is rich in fatty acids (approximately 80%), and rice oil contains unsaturated fatty acids such as oleic acid (C18:1) and α-linolenic acid (C18:3), which are essential for maintaining cell membranes and nervous system function [20]. Despite its nutritional benefits, consumers often prefer white rice due to its better texture and eating quality, which are enhanced through the polishing process. However, this process significantly reduces the nutrient content of rice [21].

The systematic evaluation of rice quality parameters has traditionally focused on specific trait categories, often examining physical properties (grain dimensions, head rice recovery) [9, 22, 23], biochemical attributes (gelatinization temperature, amylose content) [24, 25], or nutritional characteristics (protein content, micronutrients) [17, 26]. While these targeted approaches have provided valuable insights, they may not fully capture the intricate relationships among different quality parameters or their collective influence on overall grain quality. Therefore, it is essential to highlight the significance of incorporating these various quality traits to achieve a thorough understanding of rice quality, given that consumer preferences and market demands are progressively necessitating a complete evaluation of rice characteristics. The interplay among different quality parameters constitutes a notably complex dimension of rice enhancement. For example, protein content, which is essential for nutritional value, plays a significant role in determining texture and cooking properties [27]. In a similar manner, the physicochemical properties, including gelatinization temperature and gel consistency, exhibit significant correlations with cooking and eating qualities, while also revealing intricate interactions with amylose content [25, 28]. Grasping these interrelationships is crucial for the advancement of varieties that perform exceptionally well across various quality metrics. Recent research requires sophisticated analytical approaches to effectively characterize and select superior rice cultivars. While traditional evaluation methods have focused on individual trait analysis [29], the advancement of multivariate statistical techniques offers new opportunities for understanding the complex relationships among quality parameters [30]. These approaches enable the simultaneous analysis of multiple traits, providing a more nuanced understanding of varietal differences and trait associations [30].

By integrating multivariate analytical techniques to evaluate physical, cooking, nutritional, and micronutrient traits, this study aims to bridge this gap, providing a robust framework for identifying rice cultivars that optimize both nutritional and consumer-preferred qualities. This approach will support the creation of rice varieties that better meet the dual goals of health and consumer satisfaction. The objectives of this study are to: (1) comprehensively profile four rice cultivars across 27 quality-related traits, (2) analyze and quantify interrelationships among these traits using multivariate techniques, and (3) develop a robust framework for evaluating and selecting superior rice cultivars based on multiple quality criteria. This research aims to advance breeding strategies and support the development of rice varieties that better meet consumer preferences and nutritional requirements.

## Materials and methods

### Study site and rice cultivars

The research was carried out at the Rice Technology Training Center (RTTC) in Alexandria, Field Crop Research Institute, Agricultural Research Center, Egypt (31°12'N, 30°54'E), over the 2022 and 2023 growing seasons. The experimental site features a Mediterranean climate, exhibiting moderate temperatures between 20 and 32°C throughout the rice growing season. The soil exhibited a clay texture, a pH of 8.1, an organic matter content of 1.8%, and an electrical conductivity of 1.9 dS m⁻^1^. This study evaluated four rice cultivars: Giza 178, Sakha 108, Sakha Super 300, and Egyptian Yasmin. Table 1 presents the genetic pedigree and classification of these cultivars. Sakha Super 300 and Egyptian Yasmin will thereafter be referred to as Super 300 and Yasmin, respectively, in this manuscript. Certified seeds were sourced from the Rice Research Program at the Agricultural Research Center in Sakha, Kafr El-Sheikh, Egypt. The selected cultivars were chosen for their commercial significance and varied genetic backgrounds, reflecting the primary rice varieties cultivated in Egypt.

**Table 1:** Pedigree and type of four selected rice cultivars

No	Cultivars	Pedigree	Type
1	Giza 178	Giza175/Milyang 49	Indica/Japonica
2	Sakha 108	Sakha101 ∕ HR5824 ∕∕ Sakha101	Japonica
3	^a^Sakha Super 300	unknown (newly developed)	-
4	^a^Egyptian Yasmin	IR262-43–8–11 × KDML 105	Indica

^a﻿^For simplicity, the cultivars Sakha Super 300 and Egyptian Yasmin will be referred to as Super 300 and Yasmin, respectively, throughout the remainder of this manuscript

### Experimental design and field management

The study was carried out employing a randomized complete block design (RCBD) with three replications. The dimensions of each experimental plot were 5 × 4 m, totaling 20 m^2^, and included 1-m buffer zones between plots to mitigate the risk of cross-contamination. On May 15th of both seasons, seeds were sown in nursery beds, and the 25-day-old seedlings were subsequently transplanted into the experimental plots, arranged at a spacing of 20 × 20 cm, with 2–3 seedlings allocated per hill. The established agronomic practices for the cultivation of rice in Egypt were adhered to. Nitrogen fertilizer was administered in the form of urea (46% N) in three distinct applications: one-third was applied as a basal treatment, one-third during the tillering stage, and the final one-third at the panicle initiation stage, resulting in a cumulative total of 165 kg N ha⁻^1^. Basal doses of phosphorus (45 kg P₂O₅ ha⁻^1^) and potassium (57 kg K₂O ha⁻^1^) were administered. Irrigation was consistently upheld at a standing water depth of 5 cm for the duration of the growing season, continuing until two weeks prior to harvest.

### Preparation and processing of samples

Paddy rice samples were collected at physiological maturity, characterized by a grain moisture content of around 20–22%. The samples underwent homogenization through a 36-channel mixing mechanism, which effectively partitioned the samples into two equal portions, thereby ensuring representative sampling. The preliminary cleaning process involved the utilization of an air-screen cleaner with an airflow rate of 15 m^3^/min, aimed at eliminating dust, chaff, and extraneous materials before proceeding to the hulling and milling stages. A Satake grain thickness grader (model RSS1.5) equipped with perforated cylinders of diameters 1.8, 2.0, and 2.2 mm was utilized to standardize the dimensions of the grain.

Three random rough rice samples, each weighing 100 g, were collected from each plot and conditioned to achieve a moisture content of 14% using a temperature-controlled chamber set at 30°C with 65% relative humidity for a duration of 24 h. The moisture content was assessed utilizing a digital grain moisture meter (Kett Electric Laboratory, Model PB-3009). The conditioned samples underwent a cleaning and dehulling process utilizing a Satake experimental huller (THU-35A), which operated at a speed of 1000 rpm and maintained a clearance of 0.7 mm between the rollers. The hulling efficiency remained consistently above 90%, thereby guaranteeing the quality of samples for further analyses.

### Studied characters

A total of twenty-seven traits were assessed and classified into four primary categories, accompanied by comprehensive analytical methodologies as outlined below:

#### Grain physical characteristics

##### Brown Rice (%)

Samples of 150g rough rice were dehulled utilizing a Satake THU-35A testing husker, with a rubber roll clearance set at 0.7 mm and a roll speed of 1000 rpm. The procedure was carried out at ambient temperature (25 ± 2°C) with samples exhibiting a moisture content of 14%. In accordance with the methodology established by [31] as follows:

$$\mathrm{Brown}\;\mathrm{rice}\;(\%)\;=\mathrm{Weight}\;\mathrm{of}\;\mathrm{brown}\;\mathrm{rice}\;\left(\mathrm g\right)/\mathrm{Weight}\;\mathrm{of}\;\mathrm{rough}\;\mathrm{rice}\;(\mathrm g)\times100$$

##### Milling (%)

The brown rice derived from the aforementioned samples underwent milling using a Satake milling machine (model TM05) at the Rice Technology and Training Center in Alexandria. The milled rice was subsequently weighed, and calculations were performed using the following equation [32], as follows:

$$\mathrm{Milling}\;\%\;=\;\text{Weight of milled rice }(\text{g})/\text{Weight of sample paddy used}(\text{g})\times100$$

##### Broken milled rice (%)

A sample of 100g of milled rice was classified utilizing a Satake test rice grader (Model TRG05B), which features indented cylinders with hole sizes of 5.2, 4.8, and 4.0 mm. The operational parameters consisted of a sorting duration of 8 min at a cylinder speed of 40 rpm. According to the methodology outlined by [33], as follows:

$$\mathrm{Broken}\;\mathrm{rice}\;\%\;=\text{Broken grain weight }/\text{Rough rice weight}\;\times100$$

##### Grain hardness (Kg/cm^2^)

Fifty grains per sample were evaluated under standardized conditions utilizing a Kiya Seisakusho Hardness Tester (Model 3947, Tokyo, Japan) as followed by [34]. The testing apparatus was set up with a load cell capacity of 20 kg, a compression speed of 2 mm/min, a probe diameter of 2.0 mm, and the sample temperature was kept at 25 ± 1°C. For each grain sample, hardness was evaluated by measuring both the cracking force and the crushing force. Two measurements were recorded for each parameter, and the values were averaged to determine the final hardness measurement, expressed in kilograms.

##### Grain dimensions (mm)

Grain dimensions were assessed utilizing a digital grain inspector (Kett Electric Model GM20) on a sample size of 100 whole kernels. The measurement of length was conducted along the longitudinal axis with a precision of ± 0.01 mm, while width was assessed perpendicular to the length, also with a precision of ± 0.01 mm. Thickness was measured perpendicular to the width, maintaining the same precision of ± 0.01 mm. The grain morphology was determined by calculating the ratio of length to width based on these measurements.

#### Cooking and processing traits

##### Amylose (%)

The amylose content was determined using the iodine reagent method originally described by [35] and subsequently modified by [36]. The analysis was conducted by precisely weighing 100 mg of each milled rice sample.

##### Water uptake (ml water/100gm milled grains)

Water uptake at 77°C was evaluated for milled rice samples following the protocol described by [37]. The procedure involved combining 2 g of milled rice with 18 ml of distilled water in a test tube. The mixture was initially maintained at room temperature for 30 min, after which the tube was submerged in a 77°C water bath for 45 min. Following this incubation period, the tube was immediately transferred to cold water to halt the cooking process. The volume of absorbed water was calculated by subtracting the unabsorbed water from the original 18 ml volume used in the test. Water uptake measurements were then standardized and expressed as volume per 100 g of rice.

##### Kernel elongation (%)

The length of 25 whole milled rice grains was measured using a photographic enlarger. These grains were then transferred to 25mm × 100mm test tubes containing 30 ml of distilled water and allowed to stand for 30 min at room temperature. Subsequently, the test tubes were immersed in a 98°C water bath for 10 min, followed by immediate cooling in cold water until reaching room temperature. The cooking liquid was carefully decanted, and the cooked kernels were transferred to labeled Petri dishes lined with Whatman No. 1 filter paper. The samples were left to dry overnight at room temperature. The length of the cooked kernels was then measured using the same photographic enlarger. The elongation percentage was determined according to the method described by Azeez and Shafi [38], with the elongation ratio calculated using the following equation:

$$\mathrm{Grain}\;\mathrm{elongation}\;\left(\%\right)=\mathrm{Length}\;\mathrm{after}\;\mathrm{cooking}\;\left(\mathrm{LAC}\right)-\mathrm{Length}\;\mathrm{before}\;\mathrm{cooking}\;\left(\mathrm{LBC}\right)/\mathrm{Length}\;\mathrm{before}\;\mathrm{cooking}\;\left(\mathrm{LBC}\right)\times100$$

##### Cooking time (min)

Cooking time was determined following the method described by [33]. Ten whole milled rice grains were soaked in 20 mL of distilled water and placed in a water bath maintained at 98°C. The cooking process was monitored by removing and examining a grain at one-minute intervals, pressing it between two clean glass slides to check for the disappearance of the hard white core. The cooking time was recorded as the total duration from the initial grain soaking until complete cooking was achieved, as indicated by the complete gelatinization of the grain core.

##### Gel Consistency (mm)

For the analysis, precisely 100 mg of rice powder, standardized to 12% moisture content, was carefully measured and transferred into 13 × 100 mm Pyrex culture tubes (catalog number 9820). The powder was then moistened by adding exactly 0.2 ml of a solution consisting of 95% ethanol containing 0.025% thymol blue indicator. This procedure was carried out in accordance with the methodology established by [39], which provides a standardized approach for preparing rice samples for subsequent gel consistency testing.

##### Gelatinization Temperature (°C)

Gelatinization temperature was determined using the alkali spreading test. Six whole milled rice grains were placed in duplicate petri dishes containing 10 ml of 1.7% KOH solution. The samples were then incubated at 30°C for 23 h following the protocol established by [40]. After incubation, the degree of endosperm disintegration was visually assessed and scored according to a seven-point scale: scores of 1–3 indicated high gelatinization temperature (> 75°C), scores of 4–5 represented intermediate gelatinization temperature (70–75°C), and scores of 6–7 denoted low gelatinization temperature (< 70°C).

#### Nutritional components

##### Protein content (%)

Protein content was determined in brown rice samples using the standard semi-micro Kjeldahl method as described by [41]. Ammonia was captured in a 4% boric acid solution containing Tashiro indicator and subsequently titrated with 0.02 N hydrochloric acid. The nitrogen content percentage was converted to crude protein by multiplying by a factor of 5.95.

##### Fiber content (%)

Fiber content was measured by combining 2 g of sample with 0.5 g of ignited asbestos and 200 ml of 1.25% (w/v) sulfuric acid. The mixture was boiled under a reflux condenser for 30 min, then filtered through a Gooch crucible fitted with an asbestos mat and thoroughly washed with hot distilled water. The residue and asbestos were then boiled with 200 ml of 1.25% aqueous sodium hydroxide solution for 30 min and filtered again through a Gooch crucible as previously described. The residues were washed with hot water, ethyl alcohol, and acetone before drying at 110°C to constant weight. Fiber content was calculated by subtracting the ash content from the dry weight of the treated material [42].

##### Fat content (%)

Fat content was determined by extracting 2 g samples of stabilized rice bran from all investigated rice varieties with hexane in a Soxhlet apparatus for 16–18 h. The solvent was evaporated from the extract under reduced pressure, and fat content was calculated according to the methods followed by [42].

##### Ash content (%)

Ash content was determined following [43]. Bran samples (2g) were weighed into pre-ignited and pre-weighed crucibles. The samples were first charred on a hot plate, then transferred to a muffle furnace. The temperature was then gradually increased to 550°C and maintained for 8 h until a light gray ash was obtained. The samples were cooled in a desiccator and weighed. The process was repeated until constant weight was achieved, and the ash content was calculated as a percentage of the original sample weight.

##### Total carbohydrate (%)

Total carbohydrate content in defatted bran was estimated by difference using the equation:

$$\mathrm{Total}\;\mathrm{carbohydrate}\;\left(\%\right)\;=100-\left[\mathrm{Weight}\;\mathrm{in}\;\mathrm{grams}\;\left(\mathrm{protein}\;+\;\mathrm{fiber}\;+\;\mathrm{fat}\;+\;\mathrm{ash}\right)\;\mathrm{in}\;100\;\mathrm g\;\mathrm{of}\;\mathrm{bran}\right]$$

#### Minerals and vitamins

##### Mineral content (mg/kg rice)

The AOAC (2005) techniques were followed for doing mineral analysis. At 180°C, 0.5g of rice samples were digested using a 5:1 solution of concentrated HNO₃ and HClO₄ until a clear solution was produced. Deionized water was used to dilute the digested sample to 50 mL. The ascorbic acid method [44] was used to colorimetrically estimate the phosphorus (P) level. A UV–visible spectrophotometer was used to quantify the blue color intensity at 880 nm. Potassium (K) content was determined using flame photometry as described by [45]. Rice samples were dry-ashed, dissolved in dilute HCl, filtered and appropriately diluted. The solution was then atomized into a flame, causing potassium atoms to emit light at 766.5 nm. This emission intensity, directly proportional to potassium concentration, was measured and compared against a calibration curve prepared with standard solutions to calculate potassium content in mg/100g of rice (dry weight basis).

##### Vitamin content (mg/kg rice)

Vitamin analysis began with a one-minute homogenization of samples in distilled water using a speed II laboratory mill, followed by a ten-minute ultrasonic bath treatment. Samples were then filtered through a 0.45 mm PTFE paper filter. Analysis was performed using an Agilent 1200 Infinity Series HPLC with an analytical reversed-phase C-18 column (75 × 4.6 mm). Separation conditions included UV detection at 204 nm, a mobile phase of water and acetonitrile (99:1) at a flow rate of 0.5 mL/min, and room temperature analysis. Polyscience-vitamin analytical standards (USA, Illinois) were used for standard curve calibration. All measurements were conducted in triplicate [46].

### Data analysis

The experiment utilized a randomized complete block design (RCBD) with three replications across two growing seasons (2022 and 2023). Statistical analyses were conducted using RStudio (version 4.2.0, RStudio PBC, Boston, MA). Mean comparisons among cultivars were performed utilizing Tukey’s Honest Significant Difference (HSD) test with a significance level of *p* ≤ 0.05. The normality of residuals was assessed through the Shapiro–Wilk test, while homogeneity of variances was evaluated using Levene’s test [47]. Violin plots were generated using the ‘ggplot2’ package (version 3.4.0) to facilitate comparative profiling of the four rice cultivars across the 27 assessed traits. The plots integrate kernel density estimation with boxplots to depict the complete distribution of data points, median values, and quartile ranges.

Multivariate analyses were performed utilizing various complementary methods, including Pearson correlation coefficients (corrplot package v0.92), hierarchical cluster analysis (hclust function with Ward’s minimum variance method and ComplexHeatmap package v2.12.0), Principal Component Analysis (FactoMineR package v2.6 and factoextra v1.0.7), and radar plot analysis (fmsb package v0.7.5) [48]. The data were standardized for principal component analysis (PCA) and radar plots, with PCA components identified based on eigenvalues greater than 1. Correlation matrices employed hierarchical clustering, with color intensity and circle size indicating correlation strength, while heatmaps illustrated standardized Z-scores for trait-cultivar combinations. All analyses were performed at a significance level of *p* ≤ 0.05.

## Results

### Analysis of genetic and environmental components of rice quality parameters across seasons of study

Analysis of variance revealed significant genetic influences across multiple rice quality parameters (Table 2). The effects were examined across four major categories: grain physical characteristics, cooking and processing properties, nutritional components, and mineral and vitamin content.

**Table 2:** Analysis of variance for physical, biochemical, processing, and nutritional quality parameters of rice cultivars evaluated across different growing seasons

**A. Grain Physical Characteristics**							
**Source of Variance**	**Brown rice**	**Milling**	**Broken**	**Hardness**	**Grain length**	**Grain width**	**Grain thickness**
Cultivar (C)	18.9881^***†^	21.4583^***^	8.3439^***^	3.5526^***^	4.4968^***^	1.1355^***^	0.1369^***^
Season (S)	0.1734^ns^	0.0014^ns^	0.0988^ns^	0.1262^ns^	0.0590^ns^	0.0337^ns^	0.0417^**^
C x S	0.2431^ns^	0.6085^**^	0.0957^ns^	0.0874^ns^	0.0002^ns^	0.0089^ns^	0.0061^ns^
Residuals	0.0857	0.0818	0.0961	0.0998	0.0654	0.0136	0.0043
**B. Cooking and Processing Properties**							
**Source of Variance**	**Amylose**	**Water uptake**	**Elongation**	**Cooking time**	**Gel consistency**	**GT Spreading**	**GT Clearing**
Cultivar (C)	29.6419^***^	1095.935^***^	53.2738^***^	27.7213^***^	38.2305^**^	12.6905^***^	13.1641^***^
Season (S)	0.5766^*^	45.5953^*^	0.2731^ns^	0.0035^ns^	1.0086^ns^	0.0434^ns^	0.1233^ns^
C x S	0.0719^ns^	50.2150^**^	1.5826^ns^	0.5447^*^	2.0745^ns^	0.1115^ns^	0.1339^ns^
Residuals	0.1264	7.7416	2.7999	0.1264	3.2412	0.0651	0.1191
**C. Nutritional Components**							
**Source of Variance**	**Protein**	**Fiber**	**Fat**	**Ash**	**Carbohydrates**		
Cultivar (C)	1.9061^***^	0.0092^***^	0.0503^***^	0.0379^***^	3.5946^***^		
Season (S)	0.0241^ns^	0.0001^ns^	0.0026^**^	0.0020^**^	0.0038^ns^		
C x S	0.0719^ns^	0.0001^ns^	0.0000^ns^	0.0001^ns^	0.0781^ns^		
Residuals	0.0402	0.0003	0.0002	0.0002	0.1738		
**D. Minerals and Vitamins**							
**Source of Variance**	**P**	**K**	**Vitamin B1**	**Vitamin B2**	**Vitamin B3**	**Vitamin B5**	**Vitamin B6**
Cultivar (C)	5452.0^***^	2212.0^***^	0.1005^*^	0.0025^**^	61.8168^***^	3.6571^***^	1.4349^***^
Season (S)	25.6267^ns^	92.8267^*^	0.0017^ns^	0.0000^ns^	0.4401^ns^	0.0294^ns^	0.0084^ns^
C x S	172.0000^**^	82.0000^*^	0.0061^ns^	0.0001^ns^	0.1078^ns^	0.0267^ns^	0.0078^ns^
Residuals	16.1233	15.6433	0.0211	0.0003	0.2475	0.0902	0.0553

Mean square values are presented for each source of variation

*GT *Gelatinization Temperature, *P *phosphorous, *K *potassium

^†^Significance levels

*** *p* < 0.001

** *p* < 0.01

* *p* < 0.05

^ns^not significant (*P* > 0.05)

In respect to the grain physical characteristics (Table 2A), the genotypic effects were highly significant (*p* < 0.001) for all physical traits, with notably high mean squares for milling (21.46) and brown rice (18.99). The cultivar × season interaction was significant (*p* < 0.01) only for milling % (0.61). Seasonal effects were generally non-significant for physical traits, except for grain thickness (*p* < 0.01). As for cooking and processing properties (Table 2B), strong genotypic effects (*p* < 0.001) were observed for all cooking-related traits, with water uptake showing the highest mean square value (1095.94). Both season and cultivar × season interactions were significant for water uptake (*p* < 0.05 and *p* < 0.01, respectively), indicating environmental sensitivity. Amylose content exhibited significant genotypic (*p* < 0.001) and seasonal (*p* < 0.05) effects, while gel consistency showed strong genotypic effects (*p* < 0.01) but minimal environmental influence.

For nutritional components (Table 2C), all primary nutritional components displayed highly significant genotypic effects (*p* < 0.001). Fat and ash content showed significant seasonal variation (*p* < 0.01), while other nutritional parameters remained stable across seasons. The cultivar × season interaction was non-significant for all nutritional traits, suggesting consistent genotypic expression across environments. Respecting the minerals and vitamins (Table 2D), phosphorus and potassium levels demonstrated strong genotypic effects (*p* < 0.001) and significant cultivar × season interactions (*p* < 0.01 and *p* < 0.05, respectively). Vitamin content showed varying levels of genetic variance, with Vitamins B3, B5, and B6 exhibiting highly significant genotypic effects (*p* < 0.001), while Vitamins B1 and B2 showed moderate genetic influence (*p* < 0.05 and *p* < 0.01, respectively).

### Comparative analysis of physical, cooking, nutritional, and micronutrient characteristics among rice cultivars

**Fig. 1:**
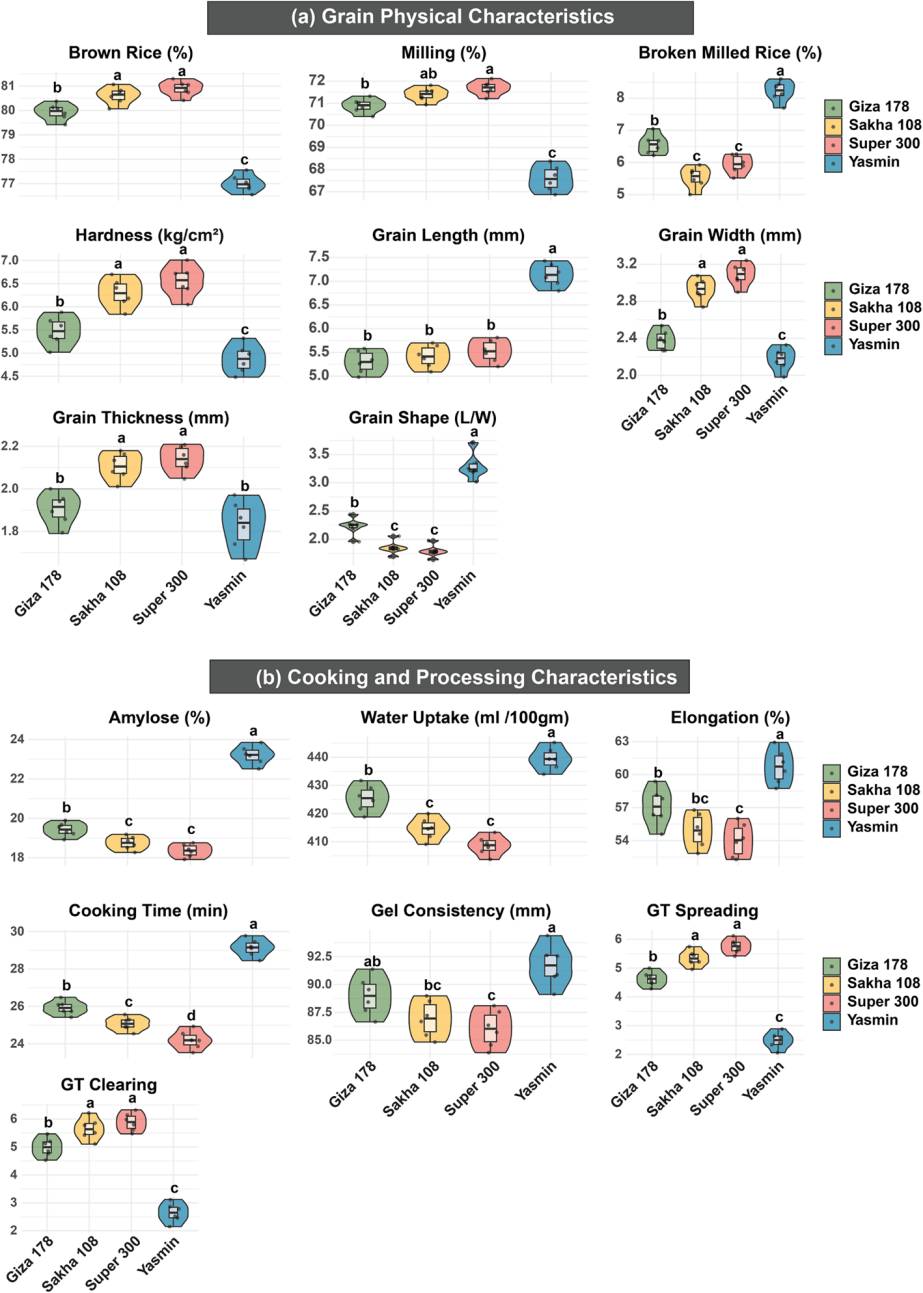
Violin plots depicting trait variation among four rice cultivars (Giza 178, Sakha 108, Super 300, and Yasmin) across key quality categories: grain physical characteristics (brown rice, milling, broken rice, hardness, grain length, width, thickness, and shape) and cooking and processing properties (amylose, water uptake, elongation, cooking time, gel consistency, GT spreading, and GT clearing). The width of each violin indicates probability density of the data distribution, with internal box plots showing median, quartiles, and extremes. Different letters above indicate significant differences among cultivars (*p* < 0.05). Color coding: Giza 178 (light green), Sakha 108 (beige), Super 300 (pink), and Yasmin (turquoise)

Analysis of grain physical characteristics (Fig. 1a) revealed distinctive patterns across the four rice cultivars, with significant variations in both milling and dimensional properties. In milling-related traits, Super 300 exhibited exceptional performance, achieving the highest values for brown rice (80.90%) and milling (71.69%), while Yasmin demonstrated notably lower values (77.02% and 67.60%, respectively). The broken rice showed an inverse relationship, with Yasmin recording the highest percentage (8.22%) and Sakha 108 maintaining the lowest (5.53%). Grain hardness displayed significant variation, with Super 300 showing superior hardness (6.56 kg/cm^2^) compared to Yasmin’s lower value (4.88 kg/cm^2^). Dimensional characteristics of rice grains revealed Yasmin’s distinctive morphology, possessing the longest grains (7.14 mm) in contrast to Giza 178’s shorter grains (5.30 mm). Also, Super 300 demonstrated superior width and thickness measurements (3.09 mm and 2.14 mm, respectively), while Yasmin showed the lowest values (2.17 mm and 1.83 mm). The grain shape ratio further emphasized these morphological differences, with Yasmin exhibiting the highest ratio (3.29) compared to Super 300’s lowest (1.79).

Regarding the cooking and processing characteristics (Fig. 1b), substantial variations among cultivars were demonstrated, with distinct patterns emerging across different parameters. Yasmin consistently exhibited superior performance in multiple cooking-related traits, recording the highest values for amylose content (23.20%), water uptake (439.45 ml 100mg^−1^), elongation (60.73%), cooking time (29.13 min), and gel consistency (91.72 mm). In contrast, Super 300 consistently showed lower values across these parameters (18.36%, 408.85 ml 100mg^−1^, 54.03%, 24.21 min, and 86.02 mm, respectively). On contrast, this pattern was reversed for gelatinization characteristics, where Super 300 demonstrated superior performance in both GT spreading (5.76) and GT clearing (5.89), while Yasmin showed significantly lower values (2.49 and 2.64, respectively). These variations suggest distinct differences in cooking behavior and end-use suitability among the cultivars.

**Fig. 2:**
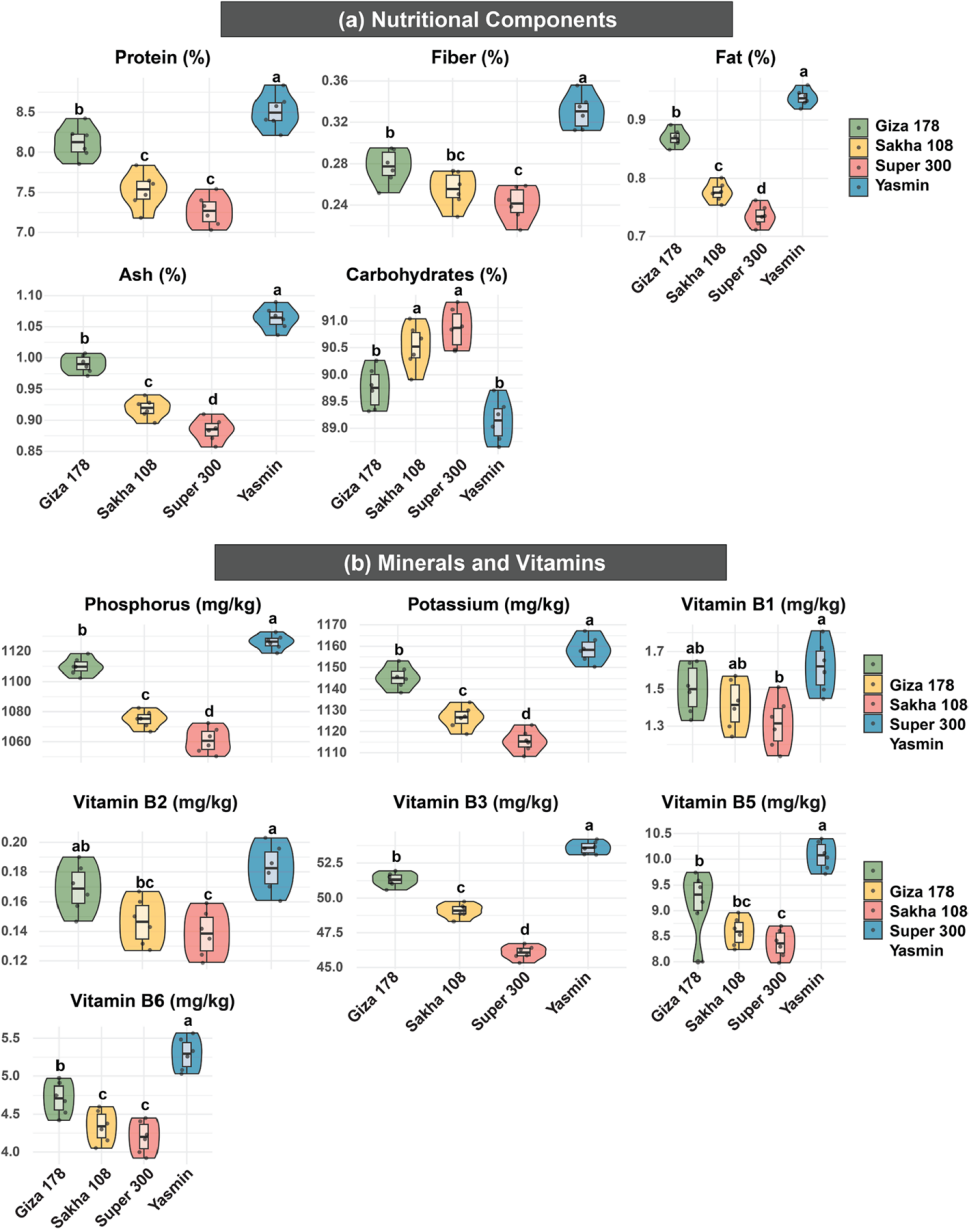
Violin plots depicting trait variation among four rice cultivars (Giza 178, Sakha 108, Super 300, and Yasmin) across key quality categories: nutritional components (protein, fiber, fat, ash, and carbohydrates), and minerals and vitamins (phosphorus, potassium, vitamins B1, B2, B3, B5, and B6). The width of each violin indicates probability density of the data distribution, with internal box plots showing median, quartiles, and extremes. Different letters above indicate significant differences among cultivars (*p* < 0.05). Color coding: Giza 178 (light green), Sakha 108 (beige), Super 300 (pink), and Yasmin (turquoise)

For the nutritional components analysis (Fig. 2a), the results revealed comprehensive differences in the nutritional profiles of the cultivars, with Yasmin demonstrating superior nutritional qualities across multiple parameters. It recorded the highest values for protein content (8.51%), fiber (0.33%), fat (0.94%), and ash (1.06%), consistently outperforming other cultivars. Super 300 consistently showed the lowest values across these nutritional parameters (7.27%, 0.24%, 0.74%, and 0.88%, respectively). An opposite pattern was recorded by carbohydrate content, with Super 300 recording the highest value (90.87%) while Yasmin showed the lowest (89.14%), which indicates significant differences in the nutritional value and potential health benefits among the cultivars.

As for minerals and vitamins content (Fig. 2b), they demonstrated marked variations, with distinct patterns emerging across different micronutrients. Yasmin exhibited superior mineral content, recording the highest values for both P (1126 mg kg^−1^) and K (1158.50 mg kg^−1^), while Super 300 showed consistently lower values (1061.00 and 1115.50 mg kg^−1^, respectively). The vitamin profile revealed similar patterns of variation, with Yasmin demonstrating superior content in most B vitamins, recording the highest values for vitamins B1 (1.62 mg kg^−1^), B2 (0.18 mg kg^−1^), B3 (51.31 mg kg^−1^), B5 (10.07 mg kg^−1^), and B6 (5.29 mg kg^−1^). Super 300 consistently showed lower values across these vitamins (1.31, 0.14, 46.08, 8.36, and 4.20 mg kg^−1^, respectively).

### Hierarchical clustering analysis of rice cultivars and their quality traits

**Fig. 3:**
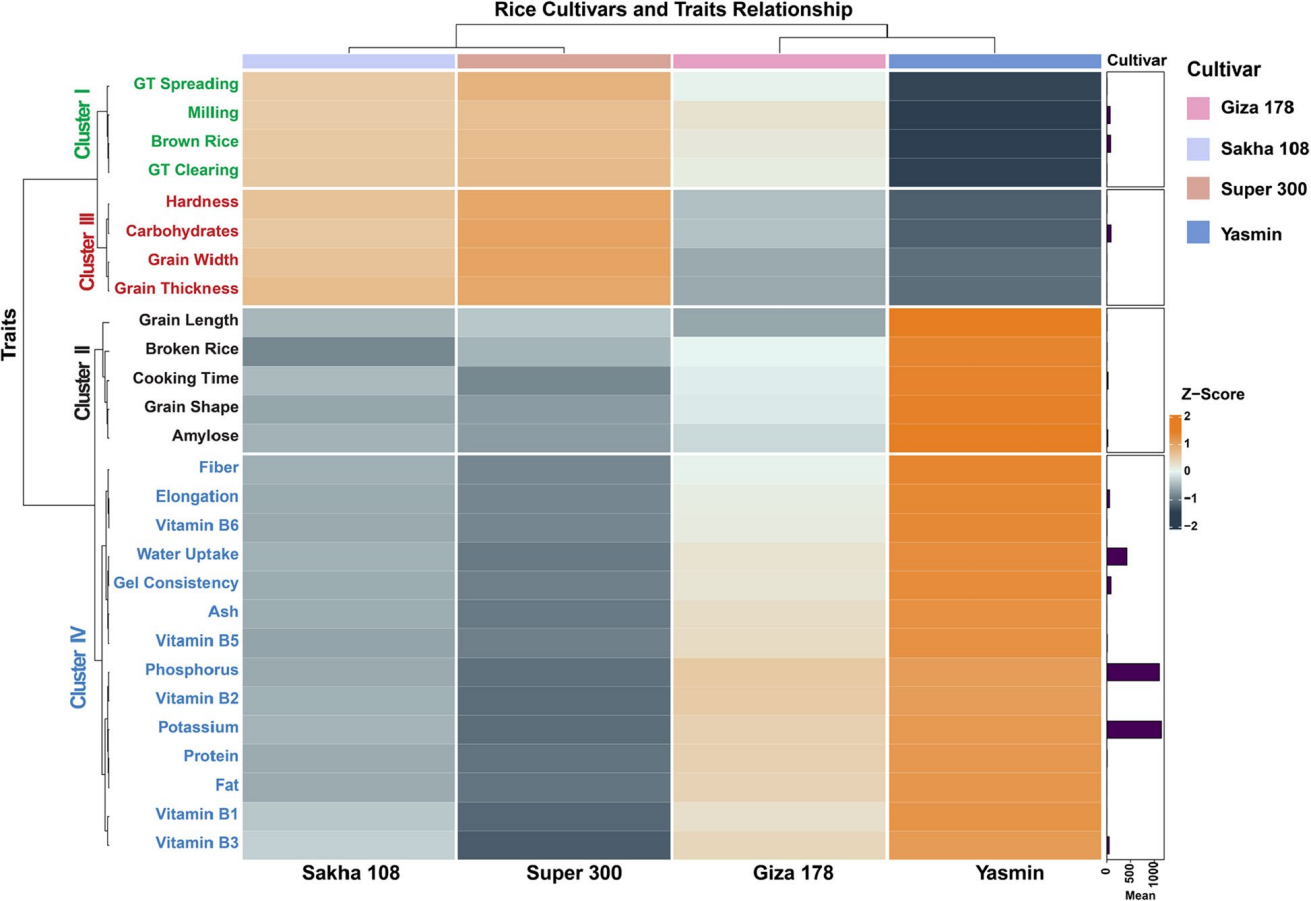
Heatmap with hierarchical clustering depicting the relationship between rice cultivars (Sakha 108, Super 300, Giza 178, and Yasmin) and 24 quality-related traits. The data is presented as Z-scores, with orange colors indicating higher values (Z-score > 0) and blue/grey colors indicating lower values (Z-score < 0) as shown in the color scale bar (ranging from −2 to + 2). The dendrogram on the left shows the clustering of traits into four distinct groups (indicated by the text colors of green (Cluster I), black (Cluster II), red (Cluster III), and blue (Cluster IV)). The top dendrogram illustrates the relationships among cultivars. The bar plot on the right represents the mean values for selected traits, highlighting significant differences among cultivars

The hierarchical cluster analysis of 27 rice quality traits revealed distinct clustering patterns at both trait and cultivar levels (Fig. 3). The trait dendrogram showed two major divisions: one predominantly containing physical and processing characteristics, and another grouping nutritional and functional properties. This hierarchical organization demonstrated the inherent relationships between quality parameters in rice, suggesting coordinated trait expression patterns. The first cluster encompassed milling-related and gelatinization characteristics, including brown rice, milling %, GT spreading, and GT clearing. These traits exhibited strong positive associations with Sakha 108 and Super 300 cultivars, indicating their superior milling and processing qualities. The second cluster comprised grain physical characteristics and cooking indicators, specifically broken rice, grain length, grain shape, amylose and cooking time. These traits showed particularly high values in the Yasmin cultivar, especially concerning grain length and shape parameters.

Physical grain properties constituted the third cluster, incorporating hardness, grain width, grain thickness, and carbohydrate content. Super 300 cultivar demonstrated superior performance across these characteristics, particularly in grain dimensional attributes. The fourth and largest cluster encompassed nutritional and functional properties, including water uptake, elongation, gel consistency, and all nutritional components (protein, fiber, fat, ash), minerals (phosphorus, potassium), and B-vitamins (B1, B2, B3, B5, B6). Yasmin cultivar exhibited remarkable superiority across this entire cluster, consistently demonstrating higher values for both nutritional and functional traits.

The cultivar dendrogram revealed two major clades: one containing Yasmin as a distinct branch, and another grouping the remaining three cultivars, with Sakha 108 and Super 300 showing closer relationship to each other than to Giza 178. This clustering pattern was supported by the trait expression profiles, where Yasmin showed distinctly superior values in nutritional and functional characteristics (demonstrated by intense orange coloration), while Sakha 108 and Super 300 shared similarities in physical and milling characteristics. Giza 178 maintained intermediate values across most traits, positioning it as a moderately performing cultivar across all quality parameters. The Z-score distribution clearly highlighted these cultivar-specific patterns, with values ranging from −2 to + 2, effectively visualizing the relative performance of each cultivar across all measured traits.

### Multidimensional analysis of rice cultivar characteristics and quality parameters

**Fig. 4:**
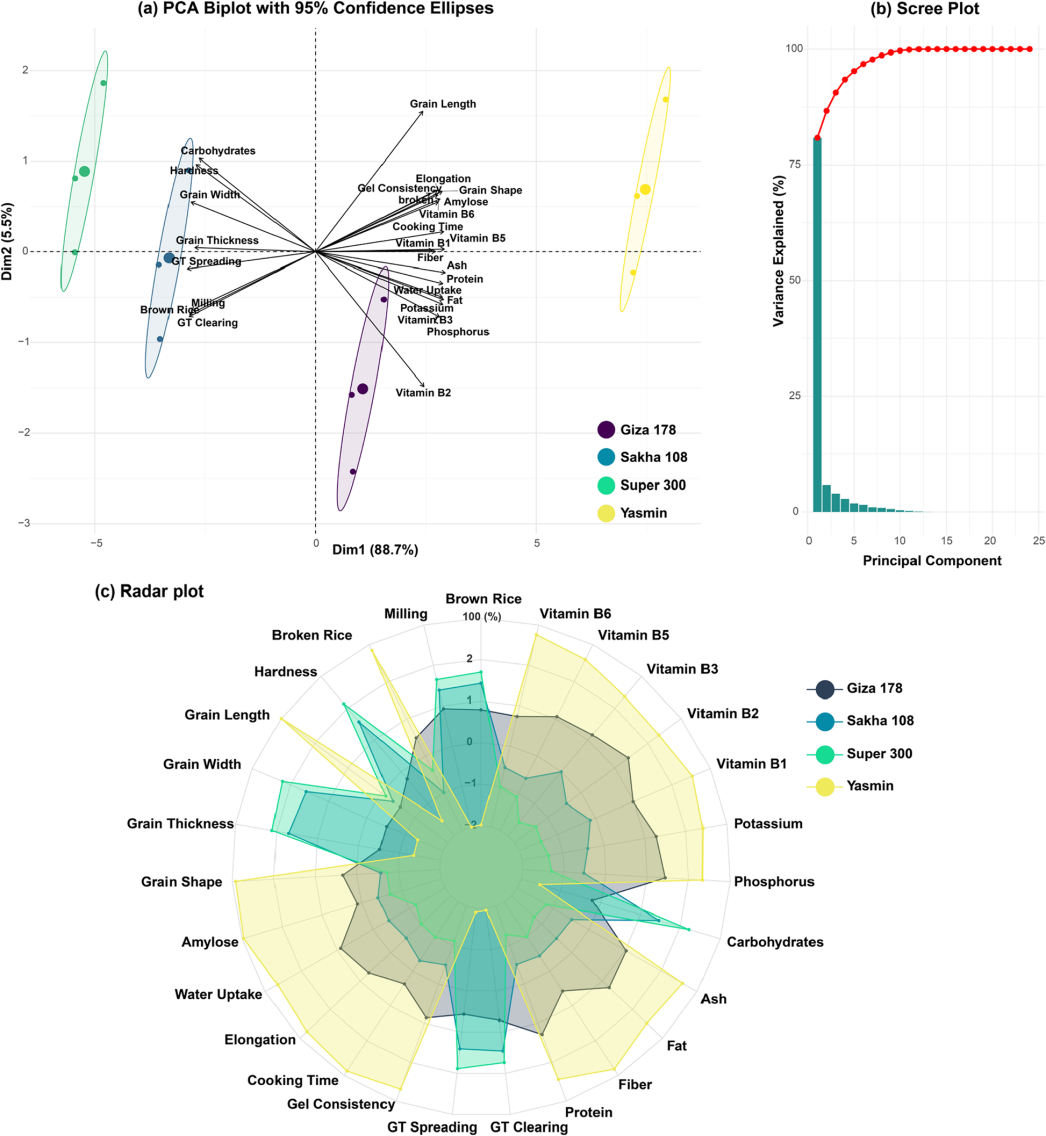
Multivariate analysis of quality parameters across four rice cultivars (Giza 178, Sakha 108, Super 300, and Yasmin). **a** PCA biplot showing the distribution of 27 quality parameters with 95% confidence ellipses. Vectors indicate the direction and strength of trait contributions, with the first two dimensions explaining 94.2% of total variance. **b** Scree plot demonstrating the percentage of variance explained by each principal component, with the first component accounting for 88.7% of total variance. **c** Radar plot visualization of 27 quality parameters illustrating the multidimensional comparison of physical, cooking, nutritional, and processing characteristics. Values are standardized on a relative scale, with colored areas representing the distinct quality profile of each cultivar

Principal Component Analysis (PCA) and multidimensional quality profiling revealed distinct patterns of variation among rice cultivars and their associated traits (Fig. 4). The first two principal components demonstrated substantial explanatory power, with Dim1 and Dim2 accounting for 88.7% and 5.5% of the total variance, respectively, collectively explaining 94.2% of the dataset variation (Fig. 4a). The scree plot analysis confirmed the predominance of these components, exhibiting a characteristic steep decline in explained variance beyond the second component (Fig. 4b).

The PCA biplot, augmented with 95% confidence ellipses, demonstrated clear cultivar differentiation across the multivariate space (Fig. 4a). Yasmin exhibited distinctive positioning in the positive quadrant of Dim1, displaying strong positive correlations with elongation, grain length, grain shape, and multiple cooking-related parameters (amylose content, cooking time, and gel consistency). Additionally, this cultivar showed significant associations with nutritional components, particularly vitamins (B1, B5, B6) and fiber content. In contrast, Super 300 and Sakha 108 clustered within the negative quadrant of Dim1, characterized by strong correlations with physical grain attributes (width, thickness) and processing parameters (GT spreading, GT clearing, milling percentage, and brown rice recovery). These cultivars demonstrated notably high factor loadings for hardness and carbohydrate content. Giza 178 occupied a distinct position in the negative quadrant of Dim2, exhibiting strong associations with mineral components (phosphorus, potassium) and specific vitamin fractions (B2, B3).

The radar plot analysis provided complementary quantitative insights into cultivar-specific quality profiles (Fig. 4c). The cultivar “Yasmin” demonstrated superior performance across multiple quality parameters, exhibiting maximum values in nutritional components (protein: 8.51%, fiber: 0.33%, fat: 0.94%, ash: 1.06%), cooking characteristics (water uptake: 439.45 mg 100g^−1^, elongation: 60.73%, cooking time: 29.14 min, gel consistency: 91.72 mm), and mineral content (P: 1126 mg kg^−1^ and K: 1158.5 mg kg^−1^). This cultivar also exhibited optimal grain length (7.14 mm) and grain shape ratio (3.29), although it demonstrated minimal values in milling properties (brown rice: 77.02%, milling: 67.60%). Super 300 exhibited contrasting characteristics, demonstrating dominance in physical and processing parameters. This cultivar showed maximum values in brown rice (80.897%), milling (71.69%), grain physical attributes (hardness: 6.56 kg cm^−2^, width: 3.09 mm, thickness: 2.14 mm), and gelatinization characteristics (GT spreading: 5.76, GT clearing: 5.89). However, it exhibited minimal values across most nutritional and cooking parameters. Sakha 108 and Giza 178 generally demonstrated intermediate values across the quality spectrum, with Sakha 108 notably exhibiting minimal broken rice (5.53%) and Giza 178 showing reduced grain length (5.30 mm).

### Comprehensive ranking analysis of four rice cultivars across grain physical characteristics, cooking and processing, nutritional components, and minerals and vitamins

**Fig. 5:**
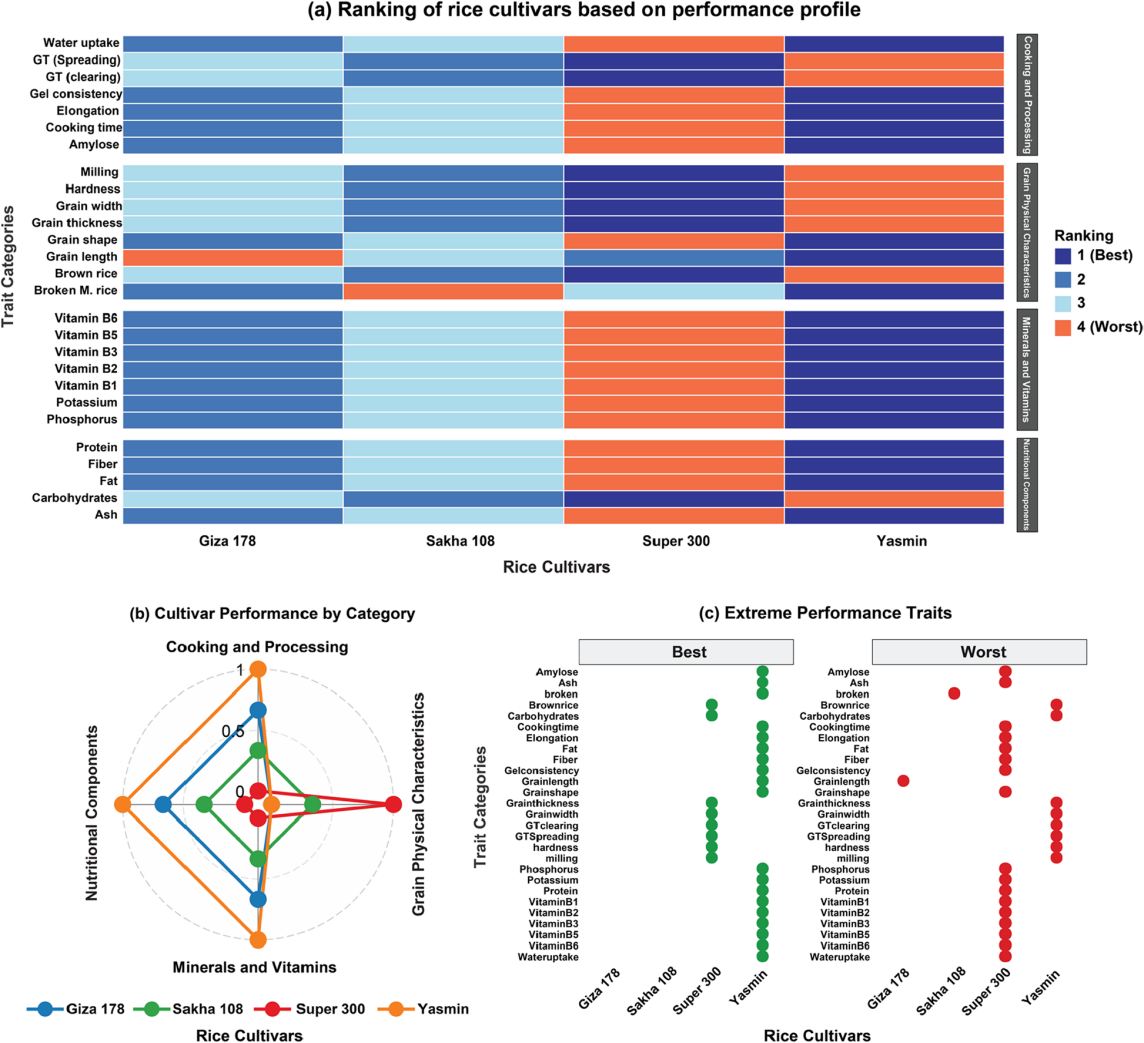
Multi-dimensional visualization comparing Giza 178, Sakha 108, Super 300, and Yasmin across 27 traits. **a** Heatmap visualization of comprehensive trait rankings organized by functional categories (Cooking and Processing, Grain Physical Characteristics, Minerals and Vitamins, and Nutritional Components); darker blue indicates superior performance (rank 1), while orange indicates inferior performance (rank 4). **b** Radar plot illustrates relative cultivar performance across the four functional categories, with larger polygon area indicating better overall ranking performance. **c** Extreme performance trait analysis displaying only the best (rank 1, in green color) and worst (rank 4, in red color) rated traits for each cultivar, revealing distinctive strength and weakness patterns

The heatmap visualization (Fig. 5a) revealed noticeable performance differentiation across the 4 rice cultivars. Yasmin demonstrated exceptional performance in cooking and processing parameters, achieving superior rankings (rank 1) in all seven traits within this category, including amylose content, water uptake, elongation, cooking time, gel consistency, whereas it showed worse ranking for the gelatinization temperature characteristics (GT spreading and GT clearing). Conversely, Super 300 consistently ranked poorest (rank 4) across these same parameters. This contrast indicates fundamentally different cooking behaviors between these cultivars, positioning Yasmin as highly suitable for applications prioritizing optimal cooking characteristics. In grain physical characteristics, an inverse performance pattern emerged, as Super 300 excelled in multiple parameters, including milling, grain thickness, grain width, and hardness (rank 1), while Yasmin performed poorly in these dimensions (rank 4). However, Yasmin demonstrated superior grain length and grain shape. Giza 178 showed moderate performance across most physical traits except grain length (rank 4), while Sakha-108 maintained consistently intermediate rankings, establishing it as a balanced option for physical grain quality. Nutritional component analysis revealed similar patterns of cultivar differentiation. Yasmin achieved superior rankings in protein, fiber, fat, and ash content, while ranking lowest in carbohydrates where Super 300 excelled. This indicates that Yasmin offers enhanced nutritional density through higher protein and micronutrient content, while Super-300 may be preferred for higher carbohydrate applications.

The radar plot analysis (Fig. 1b) illustrated the categorical performance distribution, with Yasmin demonstrating clear superiority in cooking/processing, nutritional components, and minerals/vitamins categories, while Super 300 showed strength primarily in grain physical characteristics. Giza 178 and Sakha108 exhibited more balanced performance across categories, with moderate rankings predominating. Examination of extreme performances (Fig. 1c) further confirmed these patterns, with Yasmin achieving the highest number of top rankings (21 traits at rank 1), primarily dominated by cooking, nutritional, and mineral/vitamin parameters. Conversely, Super-300 received the lowest rankings (19 traits at rank 4) in these same categories, though it excelled in specific physical characteristics.

### Correlations analysis among physical, cooking-processing, nutritional, and minerals-vitamins characteristics of rice cultivars

**Fig. 6:**
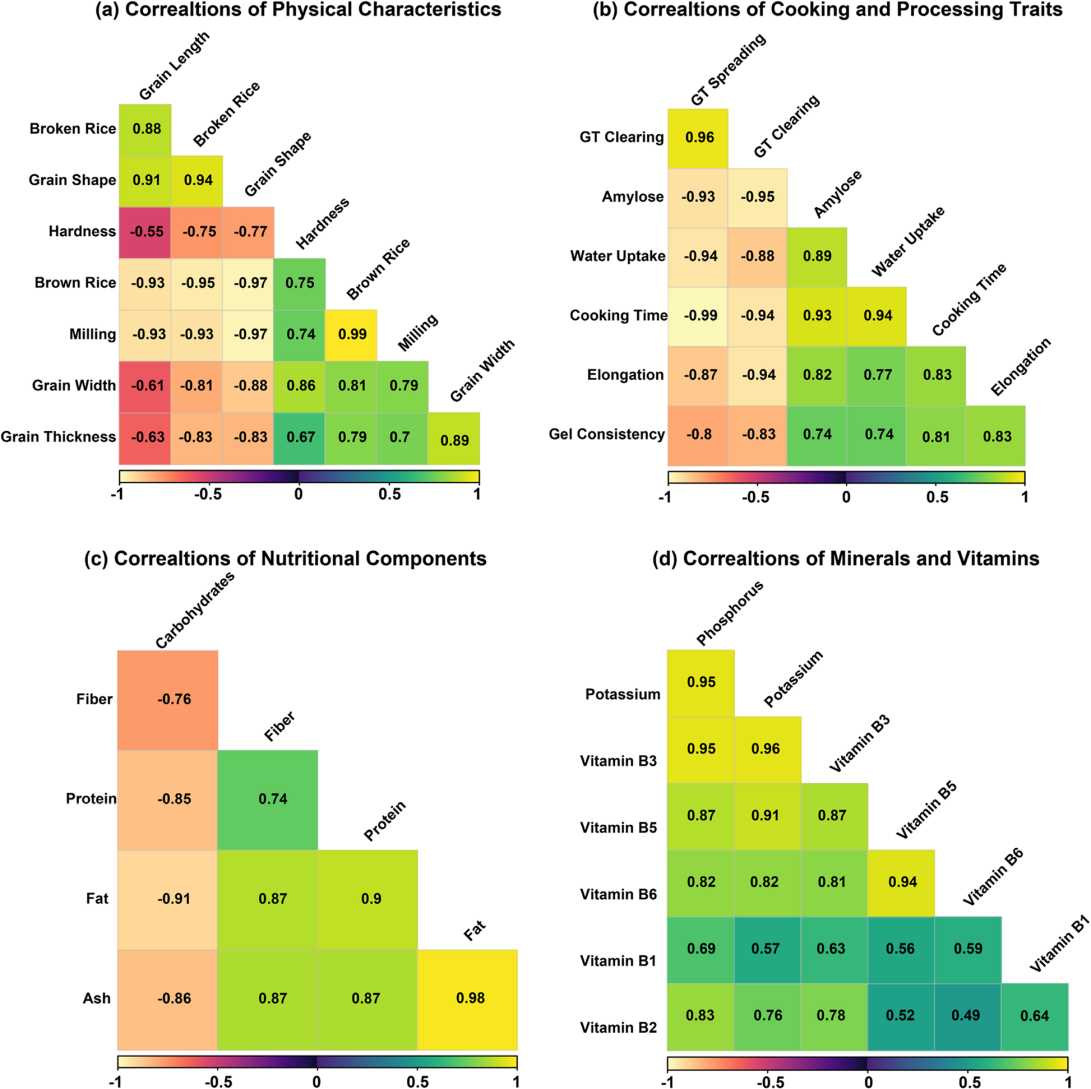
Correlation matrices showing relationships between different rice grain characteristics across four categories: **a** Physical Characteristics, **b** Cooking and Processing Traits, **c** Nutritional Components, and **d** Minerals and Vitamins. The color scale ranges from red (negative correlations) through yellow (neutral) to green (positive correlations), with values ranging from −1 to 1. The matrices reveal various strong positive and negative correlations between traits within each category, with correlation coefficients displayed in each cell

A comprehensive correlation analysis was conducted across 27 traits grouped into four categories to understand the interrelationships among various rice grain characteristics (Fig. 6). The analysis revealed significant correlations within each category, providing valuable insights for rice breeding programs.

#### Physical characteristics

The correlation analysis of physical characteristics in rice cultivars revealed strong relationships among different traits (Fig. 6a). Brown rice showed a very strong positive correlation with milling (*r* = 0.99, *p* < 0.001), and strong positive correlations with Grain width (*r* = 0.81, *p* < 0.001) and Grain thickness (*r* = 0.79, *p* < 0.001). However, it demonstrated very strong negative correlations with broken grains (*r* = −0.95, *p* < 0.001), Grain length (*r* = −0.93, *p* < 0.001), and Grain shape (*r* = −0.97, *p* < 0.001), while showing a strong positive correlation with hardness (*r* = 0.75, *p* < 0.001).

Milling characteristics displayed very strong negative correlations with broken grains (*r* = −0.93, *p* < 0.001), Grain length (*r* = −0.93, *p* < 0.001), and Grain shape (*r* = −0.97, *p* < 0.001), while showing strong positive correlations with hardness (*r* = 0.74, *p* < 0.001), Grain width (*r* = 0.79, *p* < 0.001), and Grain thickness (*r* = 0.77, *p* < 0.001). Grain hardness showed strong negative correlations with broken grains (*r* = −0.75, *p* < 0.001) and Grain shape (*r* = −0.77, *p* < 0.001), but strong positive correlation with Grain width (*r* = 0.86, *p* < 0.001) and moderate positive correlation with Grain thickness (*r* = 0.67, *p* < 0.001). Grain dimensions showed distinctive correlation patterns, with Grain length exhibiting very strong positive correlation with broken grains (*r* = 0.88, *p* < 0.001) and Grain shape (*r* = 0.91, *p* < 0.001), while showing moderate to strong negative correlations with Grain width (*r* = −0.61, *p* < 0.001) and Grain thickness (*r* = −0.63, *p* < 0.001). Grain width demonstrated strong positive correlation with Grain thickness (*r* = 0.89, *p* < 0.001) and strong negative correlation with Grain shape (*r* = −0.88, *p* < 0.001). Finally, Grain thickness showed a strong negative correlation with Grain shape (*r* = −0.83, *p* < 0.001).

#### Cooking and processing traits

In cooking and processing traits (Fig. 6b), GT clearing exhibited very strong to strong correlations with all traits, showing very strong positive correlation with GT spreading (*r* = 0.96, *p* < 0.001), and very strong negative correlations with Amylose (*r* = −0.95, *p* < 0.001), cooking time (*r* = −0.94, *p* < 0.001), and Elongation (*r* = −0.94, *p* < 0.001). Additionally, GT Clearing showed strong negative correlations with Water uptake (*r* = −0.88, *p* < 0.001) and Gel consistency (*r* = −0.83, *p* < 0.001). GT spreading demonstrated very strong negative correlations with multiple traits, particularly with cooking time (*r* = −0.99, *p* < 0.001), Water uptake (*r* = −0.94, *p* < 0.001), and Amylose (*r* = −0.93, *p* < 0.001). It also showed strong negative correlations with Elongation (*r* = −0.87, *p* < 0.001) and Gel consistency (*r* = −0.80, *p* < 0.001). Amylose content displayed a very strong positive correlation with cooking time (*r* = 0.93, *p* < 0.001), and strong positive correlations with Water uptake (*r* = 0.89, *p* < 0.001), Elongation (*r* = 0.82, *p* < 0.001), and Gel consistency (*r* = 0.74, *p* < 0.001).

Water uptake showed a very strong positive correlation with cooking time (*r* = 0.94, *p* < 0.001) and strong positive correlations with both Elongation (*r* = 0.77, *p* < 0.001) and Gel consistency (*r* = 0.74, *p* < 0.001). Cooking time demonstrated strong positive correlations with Elongation (*r* = 0.83, *p* < 0.001) and Gel consistency (*r* = 0.81, *p* < 0.001). Finally, Elongation showed a strong positive correlation with Gel consistency (*r* = 0.83, *p* < 0.001).

#### Nutritional components

The analysis of nutritional components revealed strong interrelationships among various nutrients (Fig. 6c). Protein showed strong positive correlations with Fat (*r* = 0.90, *p* < 0.001), Ash (*r* = 0.87, *p* < 0.001), and Fiber (*r* = 0.74, *p* < 0.001), while demonstrating a strong negative correlation with Carbohydrates (*r* = −0.85, *p* < 0.001). Fat content exhibited very strong positive correlations with Ash (*r* = 0.98, *p* < 0.001) and Fiber (*r* = 0.87, *p* < 0.001), and showed a very strong negative correlation with Carbohydrates (*r* = −0.91, *p* < 0.001). Additionally, Fiber demonstrated a strong positive correlation with Ash (*r* = 0.87, *p* < 0.001) and a strong negative correlation with Carbohydrates (*r* = −0.76, *p* < 0.001). Ash content also showed a strong negative correlation with Carbohydrates (*r* = −0.86, *p* < 0.001).

#### Minerals and vitamins

The analysis of minerals and vitamins revealed significant interrelationships among nutrients (Fig. 6d). Phosphorus showed very strong positive correlations with several nutrients, particularly with Vitamin B3 (*r* = 0.95, *p* < 0.001) and Potassium (*r* = 0.95, *p* < 0.001). It also demonstrated strong positive correlations with Vitamin B2 (*r* = 0.83, *p* < 0.001), Vitamin B5 (*r* = 0.87, *p* < 0.001), and moderate positive correlation with Vitamin B1 (*r* = 0.69, *p* < 0.001) and Vitamin B6 (*r* = 0.82, *p* < 0.001). Potassium exhibited very strong positive correlations with Vitamin B3 (*r* = 0.96, *p* < 0.001) and Vitamin B5 (*r* = 0.91, *p* < 0.001), while showing strong positive correlations with Vitamin B2 (*r* = 0.76, *p* < 0.001) and Vitamin B6 (*r* = 0.82, *p* < 0.001), and moderate positive correlation with Vitamin B1 (*r* = 0.57, *p* < 0.001). Among the B vitamins, Vitamin B3 showed strong positive correlations with Vitamin B5 (*r* = 0.87, *p* < 0.001) and Vitamin B6 (*r* = 0.80, *p* < 0.001), while Vitamin B5 displayed a very strong positive correlation with Vitamin B6 (*r* = 0.94, *p* < 0.001). Vitamin B1 and B2 showed moderate to strong positive correlations with each other (*r* = 0.64, *p* < 0.001) and with Vitamin B3 (*r* = 0.63, *r* = 0.78, respectively, *p* < 0.001).

## Discussion

This comprehensive investigation of rice quality parameters across physical, cooking-processing, nutritional, and mineral-vitamin characteristics in this study provides valuable insights into the complex interplay of genetic and environmental factors influencing rice grain quality.

### Genetic and environmental influences on rice quality parameters

The analysis of variance revealed predominant genetic control across most quality parameters, with notably limited environmental influence across seasons. This environmental stability in rice quality traits is particularly significant for tropical and subtropical regions where rice is commonly grown across different seasons [49, 50]. The minimal seasonal effects observed suggest that the expression of most quality traits remains consistent regardless of the growing season, a finding particularly valuable for regions with multiple rice-growing seasons per year [51].

Importantly, the strong genetic control coupled with minimal environmental interaction for most physical and nutritional traits indicates that these characteristics are inherently stable in rice [52]. This stability is crucial for maintaining consistent grain quality, a key requirement for both market acceptance and end-use applications in rice. However, the significant cultivar × season interactions observed for milling percentage and water uptake align with the known sensitivity of these traits to environmental conditions, particularly temperature and humidity during grain filling and post-harvest processing [53, 54].

Furthermore, the stability of nutritional and mineral components across seasons is particularly noteworthy for rice breeding programs aimed at biofortification [55, 56]. This environmental stability suggests that nutritional improvements achieved through breeding would likely be maintained across different growing conditions, an important consideration for addressing nutritional security through rice biofortification programs. These findings suggest that while environmental testing remains crucial for milling and water uptake characteristics, many quality parameters can be reliably selected for without extensive multi-environment trials, thereby potentially accelerating the breeding process [57–59].

### Varietal differences in rice quality characteristics

The comparative analysis in this study revealed distinct quality profiles among the studied rice cultivars, with clear trade-offs between physical processing efficiency and nutritional-cooking qualities. This differentiation highlights the importance of targeted varietal selection based on intended end-use applications in rice [60].

The current findings indicated that Super 300 exhibited superior milling and physical characteristics, with exceptional brown rice and milling percentages, coupled with higher grain hardness. These characteristics, particularly important for commercial milling operations, which suggest enhanced resistance to breakage during processing [61]. The cultivar’s superior physical processing attributes align with findings from recent studies emphasizing the economic importance of milling efficiency in rice production systems [62, 63]. Conversely, Yasmin demonstrated exceptional cooking and nutritional qualities, with superior performance in amylose content, water uptake, and elongation ratio. These characteristics, coupled with higher protein, fiber, and mineral content, position this cultivar favorably for markets prioritizing nutritional value and cooking quality [64, 65].

The micronutrient profiles reveal significant potential for nutritional improvement through varietal selection [18]. Yasmin’s superior mineral and vitamin content demonstrates the feasibility of developing rice varieties with enhanced nutritional value without compromising essential cooking qualities, supporting current biofortification efforts in rice breeding programs. Such clear differentiation obtained in the current investigation in quality profiles emphasizes market-specific varietal development as crucial for effective breeding programs.

### Multivariate analysis reveals distinct quality profiles and trait associations in rice cultivars

The multivariate analyses provided nuanced insights into the complex interrelationships of rice quality traits across different cultivars. By utilizing hierarchical clustering, PCA, and radar plot analysis, we identified two fundamental dimensions that characterize rice quality: physical-processing attributes and nutritional-functional properties. This classification reveals critical genetic and phenotypic connections that have significant implications for rice breeding strategies [66]. The clustering pattern demonstrated a meaningful organization of traits, with milling-related characteristics closely associated with gelatinization properties, while nutritional components aligned with cooking attributes [67]. This suggests the underlying genetic mechanisms that coordinate related quality traits.

The clear differentiation among cultivars, particularly the unique positioning of Yasmin, provides valuable insights for targeted parental selection in breeding programs [68]. Substantiating these relationships, PCA with the first two components explaining a substantial proportion of trait variance [69]. These distinct quality profiles highlighted a critical trade-off in rice breeding: cultivars like Yasmin excel in nutritional and cooking properties, while others like Super 300 demonstrate superior physical processing characteristics. This underscores the complexity of developing rice varieties with optimal overall quality. In this concern, rather than pursuing a universal high-quality variety, breeding programs should focus on developing cultivars tailored to specific market segments and end-use applications [70]. The observed trait associations within clusters indicate potential for simultaneous improvement of related characteristics through precise genetic selection. Moreover, even the intermediate positioning of Giza 178 and Sakha 108 suggests these cultivars may serve as valuable genetic resources for developing varieties with more balanced quality profiles.

The multivariate analysis provides a comprehensive framework for understanding the intricate relationships between rice quality traits, offering guidance for more targeted and efficient breeding strategies. These insights contribute to the broader rice research literature by demonstrating the complex interplay between genetic variation and quality characteristics [71], ultimately supporting more sophisticated approaches to rice variety development.

### Integrated quality correlation framework reveals the interdependencies of rice traits

The correlation analysis across the four trait categories in the current study provides a sophisticated framework for understanding the complex interrelationships that define rice quality and technological potential. By examining the intricate connections among physical, cooking-processing, nutritional, and mineral-vitamin characteristics, our research reveals significant insights into rice cultivar optimization. This comprehensive analysis exceeds traditional single-trait approaches, offering a holistic framework for rice quality assessment. By mapping the intricate correlations across physical, technological, nutritional, and mineral dimensions, our study establishes a robust scientific foundation for precision breeding and advanced rice quality management strategies. The findings highlight the complexity of rice quality, demonstrating that cultivar improvement requires a multidimensional approach that considers the nuanced interactions among various trait categories. Recent studies have shown how multivariate analyses can effectively integrate multiple traits to enhance crop breeding efficiency [72, 73].

The physical traits correlations reveal critical insights into grain morphology and processing efficiency [74, 75]. Brown rice and milling characteristics demonstrate an exceptionally strong relationship, indicating the fundamental connection between grain structure and processing potential [76]. Grain dimensions exhibit complex interactions, with length showing significant correlations with broken grains and grain shape [77]. Furthermore, cooking and processing characteristics such as gelatinization temperature traits emerged as key indicators of rice cooking properties. GT clearing demonstrated remarkable correlations across multiple cooking-related characteristics [78]. The interactions between gelatinization temperature, amylose content, and cooking time provide crucial insights into the technological parameters that determine rice cooking quality and texture [79, 80]. This analysis reveals a sophisticated interplay between various cooking and processing traits, highlighting the complex mechanisms that influence rice preparation, texture, and culinary performance.

Also, the nutritional profile uncovered significant metabolic interdependencies among protein, fat, ash, fiber, and carbohydrates. These correlations suggest intricate biochemical relationships that have substantial implications for understanding the nutritional composition of rice cultivars [81]. The observed interactions demonstrate the complex nature of nutritional components; changes in one nutrient can significantly impact the overall nutritional profile [82, 83]. This insight is crucial for breeding programs aiming to develop rice varieties with optimized nutritional characteristics. Importantly, the mineral and vitamin analysis revealed complex nutrient interactions—particularly among phosphorus potassium—and B-vitamin complexes—highlighting a sophisticated network of micronutrient relationships within rice cultivars [84, 85]. Such correlations provide valuable insights into biochemical mechanisms contributing to overall nutritional value. For instance, studies on micronutrient biofortification have shown how enhancing certain minerals like zinc or iron can improve human health outcomes when consumed through staple crops like [86, 87].

### Limitations of the study

Although our study offers extensive insights into the characteristics of rice quality, it is essential to recognize several inherent limitations. The geographical specificity of the study, carried out within a distinct regional context, may limit the overall applicability of our findings. Also, although our analysis identified notable correlations across various trait dimensions, the exact genetic mechanisms that govern these intricate trait interactions are still not fully elucidated, highlighting the need for additional molecular-level research. These limitations do not diminish the study’s scientific value but rather highlight critical areas for future research, offering a clear pathway for subsequent scientific inquiries into rice quality characterization and breeding strategies.

## Conclusions

This comprehensive evaluation of four rice cultivars (Giza 178, Sakha 108, Super 300, and Yasmin) across 27 quality traits revealed significant patterns of variation with important implications for rice breeding. The study demonstrated distinctive cultivar-specific strengths, with Yasmin exhibiting superior nutritional and functional properties (highest protein at 8.51% and fiber at 0.33%), while Super 300 excelled in physical grain attributes and milling efficiency (especially, brown rice and milling percentage. Meanwhile, Giza 178 and Sakha 108 showed balanced intermediate performance across parameters, with Sakha 108 particularly maintaining low broken rice percentage. The correlation analysis revealed significant relationships between quality parameters, particularly strong positive correlations between nutritional components and cooking properties, and between physical characteristics and milling efficiency. Principal Component Analysis showed that 94.2% of the total variance could be explained by the first two components, with clear separation of cultivars based on their quality profiles. These findings provide valuable insights for rice breeding programs and cultivar selection, demonstrating the complex relationships between various quality traits and the current trade-offs inherent in existing cultivars.

## Data Availability

All data generated or analyzed during this study are included in this published article. The datasets used and analyzed during the current study are available from the S.F.L and A.M.A. on reasonable request. No datasets were generated or analyzed during the current study.
